# Arthrodesis of the distal interphalangeal joint: an accurate method of screw length measurement

**DOI:** 10.1308/rcsann.2025.0039

**Published:** 2025-06-17

**Authors:** L Ibrahim, T Ammari, N Sheppard

**Affiliations:** Norfolk and Norwich University Hospitals NHS Foundation Trust, UK

Headless compression screws provide stable fixation in distal interphalangeal joint (DIPJ) fusion.^1,2^ This involves decortication of the articular surfaces of the distal (P3) and middle phalanx (P2) through an open surgical approach, followed by retrograde K-wire insertion and siting the screw to fuse the DIPJ.^3^ Precise screw length measurement is imperative to avoid iatrogenic injury to the proximal interphalangeal joint (PIPJ). We present a novel method for screw length measurement in DIPJ arthrodesis.

**Figure 1:**
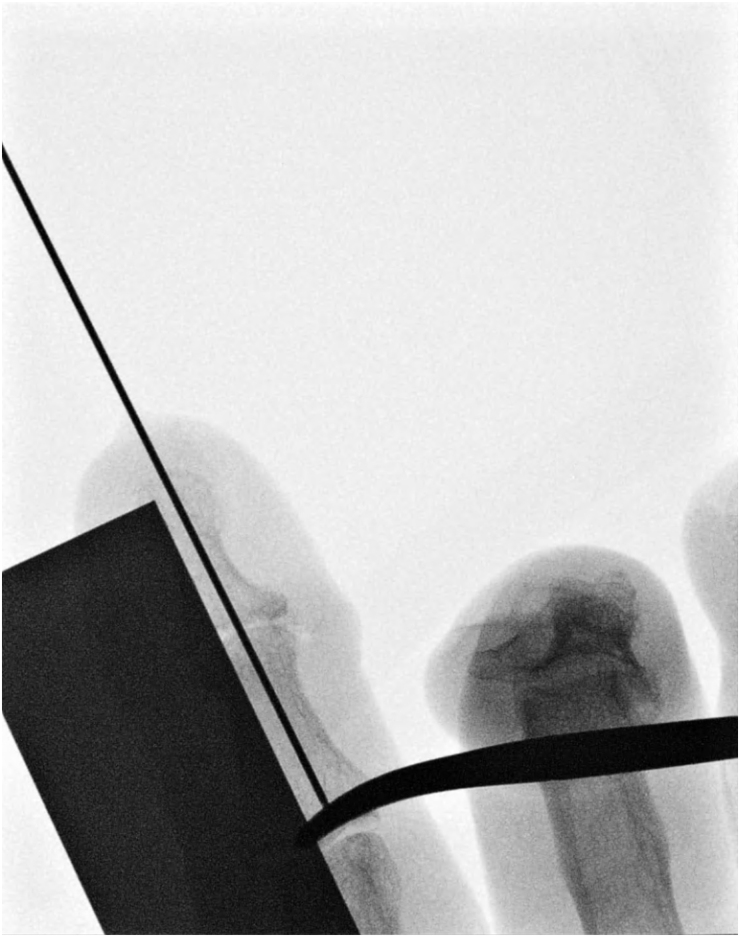
X-ray image of K-wire length measurement using Sawtell forceps and a stainless-steel ruler.

**Figure 2:**
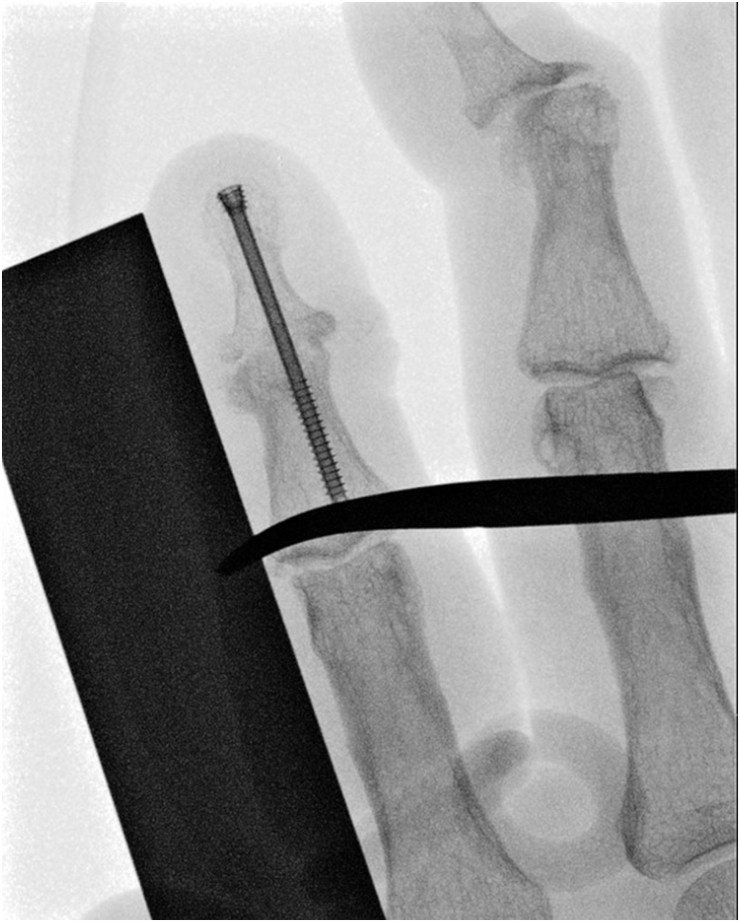
X-ray image of headless compression screw accurately positioned through the distal interphalangeal joint of the right index finger.

Traditionally, following advancement of the K-wire through the DIPJ, a depth-gauge is used to measure the length of wire protruding from the digit, determining the screw length required. An alternative is a screw depth-gauge with its own protruding wire or hook. We present another method for measurement (Figures 1 and 2), using a haemostat and a metal ruler. The haemostat is aligned with the wire tip under fluoroscopic guidance, and the required screw length can be determined from the ruler. The screw position was planned to ensure completely burying the head within the P3 tuft. The screw length was chosen to allow positioning of the threads within the narrowest part of the intramedullary canal of P2, without breaching the PIPJ. This optimises DIPJ compression while maintaining screw containment within the bony architecture.

A wire-based depth-gauge is often impaired from reaching bone by pulp septations, which warrant further dissection for accurate measurement. The conventional depth-gauge has no implement to keep in position, necessitating removal of the wire to utilise. We present a simple method of screw length measurement which can be reproduced with simple equipment.
